# EPG-5 regulates TGFB/TGF-β and WNT signalling by modulating retrograde endocytic trafficking

**DOI:** 10.1080/15548627.2025.2485420

**Published:** 2025-04-03

**Authors:** Chongzhen Yuan, Huachuan Dong, Chunyan Wu, Jinyang Liu, Zheng Wang, Xingwei Wang, Haiyan Ren, Zhaoyu Wang, Qun Lu

**Affiliations:** a State Key Laboratory of Biomacromolecules, Institute of Biophysics, Chinese Academy of Sciences, Beijing, China; b Center for Life Sciences, School of Life Sciences, Yunnan Key Laboratory of Cell Metabolism and Diseases, Yunnan University, Kunming, Yunnan, China

**Keywords:** Autophagy, *epg-5*, retrograde recycling, TGFB, WNT

## Abstract

The Vici syndrome protein EPG5 acts as a tethering factor determining the fusion specificity of autophagosomes with late endosomes/lysosomes. Here we demonstrated that during *C. elegans* development, EPG-5 modulates SMA and MAB TGFB/TGF-β signaling in controlling body size and also WNT signaling in regulating cell migration. EPG-5 is required for retrograde trafficking of the TGFB receptor SMA-6 and WLS/Wntless homolog MIG-14. In *epg-5* mutants, SMA-6 and MIG-14 are trapped within hybrid endosomal structures, which colocalize with SNX-1- and SNX-3-labeled vesicles, respectively. Basolateral recycling processes of transmembrane cargos H.s.TFR/hTfR and H.s.IL2RA/hTAC are also defective in *epg-5* mutants. Depletion of EPG-5 causes defective RAB-5 and RAB-7, and RAB-5 and RAB-10 conversion, leading to the formation of these hybrid vesicles. The defects in endocytic trafficking and autophagy in *epg-5* mutants are ameliorated by knocking down components of the HOPS complex. Our study demonstrates the intersection between the autophagy pathway and the endocytic pathway, providing insights into the pathogenesis of amyotrophic lateral sclerosis (ALS) and Vici syndrome.

**Abbreviations:** ALM: anterior lateral microtubule; ATG: autophagy related; AVM: anterior ventral microtubule; CORVET: class C core vacuole/endosome tethering; DAF-4: abnormal dauer formation 4; DIC: differential interference contrast; EPG: ectopic PGL granules; EPG-5: ectopic P granules 5; GAP: GTPase activating protein; GFP: green fluorescent protein; HOPS: homotypic fusion and vacuole protein sorting; H.s.IL2RA/hTAC: human interleukin 2 receptor subunit alpha; H.s.TFR/hTfR: human transferrin receptor; L1/L4: the first/fourth larval; mCh: mCherry; MIG-14: abnormal cell migration 14; PLM: posterior lateral microtubule; PVM: posterior ventral microtubule; RAB: ras-related protein; RFP: red fluorescent protein; RME-1: receptor mediated endocytosis 1; SMA-6: small 6; SNARE: soluble N-ethylmaleimide-sensitive factor attachment protein receptor; SNX: sorting nexin; TBC-2: TBC1 (Tre-2/Bub2/Cdc16) domain family 2; TGFB/TGF-β: transforming growth factor beta; TGN: trans-Golgi network; VPS: related to yeast vacuolar protein sorting factor; WT: wild type

## Introduction

Membrane trafficking involves the transport of proteins and lipids among membrane-bound compartments, including the plasma membrane, endoplasmic reticulum, Golgi network, autophagosomes, endosomes, and lysosomes. The coordinated action of endocytosis, secretory pathways, exocytosis, and autophagy maintains the composition of the plasma membrane and proper cellular homeostasis.

Autophagy is a lysosome-mediated degradation process which is evolutionarily conserved. It involves the formation of a double-membrane phagophore and autophagosome, and delivery of sequestered materials to the lysosome for degradation [1]. A set of *ATG* (autophagy related) genes and *EPG* (ectopic PGL granules) genes have been genetically identified in yeast and worms that are essential for autophagosome formation [2,3]. In multicellular eukaryotes, nascent autophagosomes undergo maturation processes, involving fusion with vesicles originating from the endolysosomal compartment, before forming degradative autolysosomes. The fusion of autophagosomes with endosomes/lysosomes, as with other vesicle trafficking pathways, requires the concerted actions of RABs, tethers, and soluble N-ethylmaleimide-sensitive factor attachment protein receptor (SNARE) proteins [4]. Not surprisingly, genes involved in endosome formation, maturation, and fusion play important roles in autophagy. For example, RAB7, which is essential for maturation of early endosomes to late endosomes, is also required for the final maturation of late autophagic vacuoles [5,6]. The loss of the coat protein I/COPI coatomer complex impairs the normal function of ER-Golgi transport and early endosome, as well as blocking autophagosome maturation [7]. Meanwhile, a defective autophagy pathway also impairs endocytic trafficking. The enlarged lysosomal structures in mutants resulting from mutations of lysosomal channel protein CUP-5 (coelomocyte uptake-defective 5) are ameliorated by loss of autophagy [8].

Membrane trafficking is involved in the delivery of ligands and receptors into distinct membrane-bound compartments and thus is crucial for signal transduction. After internalization, cargoes, such as receptors on the cell membrane, that enter early endosomes can be trafficked into the lysosome for degradation or recycled back to the plasma membrane through recycling endosomes or retrograde transport pathway. It has been well-known that intracellular membrane trafficking plays important roles in the TGFB/TGF-β (transforming growth factor beta) and WNT signaling pathways, which control multiple developmental processes in diverse organisms [9]. Upon ligand binding, TGFB receptors are endocytosed and initiate signaling through activating the SMAD pathway. ZFYVE9/SARA (zinc finger FYVE-type containing 9), HGS/Hrs (hepatocyte growth factor-regulated tyrosine kinase substrate), and ZFYVE16/endofin positively control the TGFB signaling levels on early/sorting endosomes. In the absence of ligand, TGFB receptors are continually endocytosed into early endosomes and recycled back to the plasma membrane [10–12]. In *C. elegans*, SMA (Small) and MAB (Male abnormal) TGFB-related signaling controls body size and male tail morphogenesis [13]. In this pathway, the binding of the DBL-1 (DPP/BMP-like 1) ligand to type I receptor SMA-6 and type II receptor DAF-4 (abnormal dauer formation 4) transduces signals into the nucleus via SMADs and regulates the transcription of target genes. SMA-6 is then recycled to the plasma membrane via the retromer complex, while DAF-4 is recycled in a retromer-independent manner mediated by ARF-6 (ADP-ribosylation factor homolog 6) [14]. Retromer is an evolutionarily conserved multimeric complex [15]. The *C. elegans* retromer consists of a cargo-recognition trimer VPS-35 (related to yeast vacuolar protein sorting factor 35)-VPS26-VPS29 and an SNX (sorting nexin)-BAR heterodimer (SNX-1 and SNX-6) or other SNX protein such as SNX-3 which lacks a BAR domain [16,17]. The canonical WNT pathway regulates the transcription of target genes via the effector protein CTNNB1/β-catenin [18]. In *C. elegans*, WNT signaling regulates diverse developmental processes, including cell migration, polarity, axon growth and branch formation, and asymmetric division of somatic gonadal precursor cells [19–23]. After synthesis, WNT is transported to the Golgi network, where the transmembrane protein MIG-14/WLS/Wntless binds to WNT and chaperones it to the cell surface for secretion [24]. MIG-14/WLS/Wntless is then endocytosed and retrogradely transported to the *trans*-Golgi network (TGN) for reuse in a SNX-3-dependent retromer pathway [17,25]. The endomembrane trafficking process of MIG-14/Wntless is essential for WNT signal transduction.

*epg-5* was identified in *C. elegans* genetic screens as a metazoan-specific autophagy gene that is required for the maturation of autophagosomes into degradative autolysosomes [26]. Loss of function of EPG5/EPG-5 causes accumulation of non-degradative autolysosomes. Human genetic studies showed that recessive mutations in *EPG5* cause Vici syndrome, a multisystem disorder characterized by agenesis of the corpus callosum, hypopigmentation, myopathy, and combined immunodeficiency [27]. The *epg5* knockout mice show cellular defects, including SQSTM1/p62 (sequestosome 1) aggregates accumulation and ubiquitin-positive inclusions formation in neurons and glial cells, along with the key features of Vici syndrome [28,29]. EPG5/EPG-5 functions as a RAB7 effector and localizes on the late endosome and lysosome. EPG5 recognizes autophagosomes and amphisomes by interacting with MAP1LC3/LC3/Atg8 (microtubule associated protein 1 light chain 3) and facilitates the assembly of STX17-SNAP29-VAMP7 or VAMP8 trans-SNARE complex for fusion with late endosomes/lysosomes. In cells depleted of *EPG5*, autophagosomes also nonspecifically fuse with other vesicles including recycling endosomes, resulting in the formation of promiscuous hybrid vesicles due to the assembly of non-physiological STX17-SNAP25-VAMP8 SNARE complexes [30]. Besides the function in autophagy, loss of *Epg5* activity also delays endocytic recycling and impairs endocytic degradation of the EGFR (epidermal growth factor receptor) [28]. However, the underlying molecular role of EPG5/EPG-5 in endocytic trafficking and related physiological processes is unclear.

Here we demonstrate that EPG-5 regulates the retrograde trafficking of TGFB receptor SMA-6 and WNT-binding protein MIG-14/Wntless, thus modulating physiological TGFB and WNT signaling in *C. elegans*. The basolateral recycling processes of transmembrane cargo proteins H.s.TFR/hTfR and H.s.IL2RA/hTAC are also defective in *epg-5* mutants. EPG-5 functions upstream of the GTPase activating protein (GAP) TBC-2 (TBC1 (Tre-2/Bub2/Cdc16) domain family 2) in the sorting or maturation of sorting/early endosomes through modulating RAB-5 and RAB-7, and RAB-5 and RAB-10 conversion, both of which depend on the GAP activity of TBC-2. Our results indicate that EPG-5 modulates vesicle-trafficking pathways to control various signaling processes under physiological conditions.

## Results

### epg-5 *mutants exhibit short body size associated with impaired TGFB signaling*

**Figure 1.**
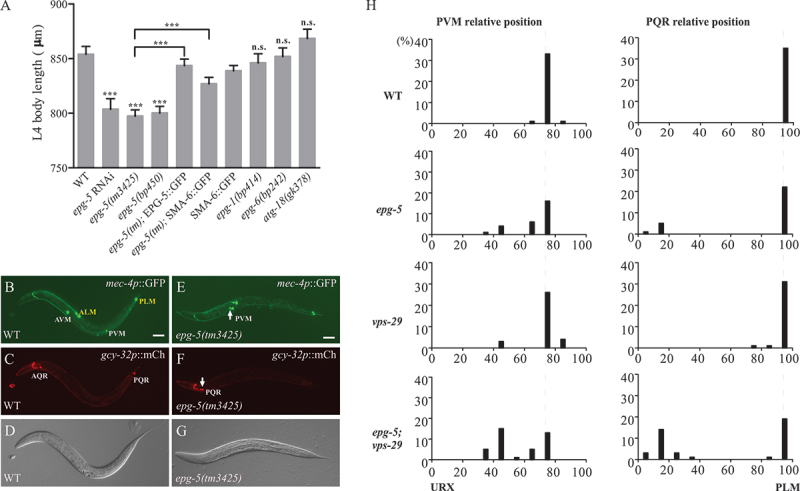
The *epg-5* mutant worms exhibit defects associated with impaired TGFB and WNT signaling. (A) Quantification of body length of L4 larvae (*n* ≥ 20) in different genetic backgrounds. L4 body length in worms with loss of function of *epg-5* show a shortened body size, while *epg-1(bp414)*, *epg-6(bp242)*, and *atg-18(gk378)* mutants have no body size change. Transgenesis using EPG-5::GFP and SMA-6::GFP could both rescue the defective body size in *epg-5(tm3425)* mutants. (B-D) The final position of Q cells descendants labelled by *mec-4p*::GFP and *gcy-32p*::mCherry in WT worms. (E-G) The final position of Q cells descendants labelled by *mec-4p*::GFP and *gcy-32p*::mCherry in *epg-5(tm3425)* mutants, which show a defective anterior localization of PVM neurons labelled by *mec-4p*::GFP and PQR neurons labelled by *gcy-32p*::mCherry. (H) Distribution chart showing the quantification of the migration of PVM and PQR neurons in WT, *epg-5(tm3425)* mutants, *vps-29(tm1320)* mutants, and *epg-5(tm3425); vps-29(tm1320)* mutants. The relative positions of PVM and PQR (*n* ≥ 28) are determined according to the strategy in Figure S1-B. Bars on the y-axis indicate the percentage of cells at each position. Dashed lines indicate the WT position. Scale bars: 50 μm.

Worms with a loss of function of core autophagy genes, including *atg-13/epg-1* (Atg13 homolog in *C. elegans*), *epg-6* (WDR45 and WDR45B homolog in *C. elegans*), and *atg-18* showed a normal body size. However, *epg-5* mutant animals (by RNAi, deletion mutation *tm3425*, and point mutation *bp450*) showed a short body size (Figure 1A). This defect was rescued by a transgene carrying EPG-5::GFP driven by its own promoter as shown before [26] or by the intestine specifically expressed promoter (Figure 1A, S1C). Body size in *C. elegans* is regulated by SMA and MAB TGFB signaling, which is composed of DBL-1 (ligand), DAF-4 and SMA-6 (receptors), and SMA-2, SMA-3, and SMA-4 (SMADs) [31]. As shown before, hypodermal expression of SMA-6 maintains the worm body length [32], it also rescued the short body length of *epg-5* mutants (Figure S1C). Surprisingly, we found that a SMA-6:GFP transgene which is specifically expressed in the intestine, but not DAF-4::GFP, partially rescued the shortened body size in *epg-5* mutants (Figure 1A, S1C). In *C. elegans*, morphogenesis of the male tail is also controlled by SMA and MAB TGFB signaling. This includes the development of male-specific sensory rays accompanied by the specification of dopaminergic (CAT-2::YFP) and serotonergic (TPH-1::CFP) ray neurons and the morphogenesis of copulatory spicules [33,34]. By quantification of CAT-2 and TPH-1 labeled ray location, there are no defects of male tail morphogenesis in *epg-5* mutants (Figure S1A1-A5, B1-B6). These results suggest that SMA and MAB TGFB signaling may be impaired in *epg-5* mutants resulting in the shorter body length.

### epg-5 *mutants exhibit defects in migration of Q* *cell descendants controlled by EGL-20/WNT signaling*

During the postembryonic stages in *C. elegans*, the two Q neuroblasts, Q neuroblast on the left side (QL) and Q neuroblast on the right side (QR), and their descendants undergo long-range migration along the anterior-posterior (A/P) body axis during the first larval stage, a process controlled by EGL-20/WNT signaling [35]. QR cell descendants (QR. d) anterior ventral microtubule cell (AVM), anterior QR descendant (AQR), and subdorsal QR descendant (SDQR) migrate anteriorly, while QL cell descendants (QL. d) posterior ventral microtubule cell (PVM), posterior QR descendant (PQR), and subdorsal QL descendant (SDQL) migrate posteriorly [36]. Loss of EGL-20/WNT leads to abnormal migration of QR. d toward the anterior [37]. The anterior migration of QR. d (AVM and AQR) and posterior migration of QL. d (PVM and PQR) were examined and we found that in *epg-5* mutants, the final position of some QL. d PVM neurons (labeled by *mec-4p*::GFP) or PQR neurons (labeled by *gcy-32p*::mCherry) showed an abnormal anterior shift (Figure 1B–H, S1D-F). *vps-29*, encoding one subunit of the retromer subcomplex, is involved in retrograde transport of MIG-14 [25]. The defect in migration of Q cell descendants was not evident in *vps-29(tm1320)* mutants. However, simultaneous depletion of *vps-29* greatly enhanced the defect in *epg-5* mutants (Figure 1H, S1E-F). These results indicate that EPG-5 functions in the WNT-dependent process of posterior migration of QL descendants.

### EPG-5 is required for retrograde endocytic trafficking of SMA-6 and MIG-14

**Figure 2.**
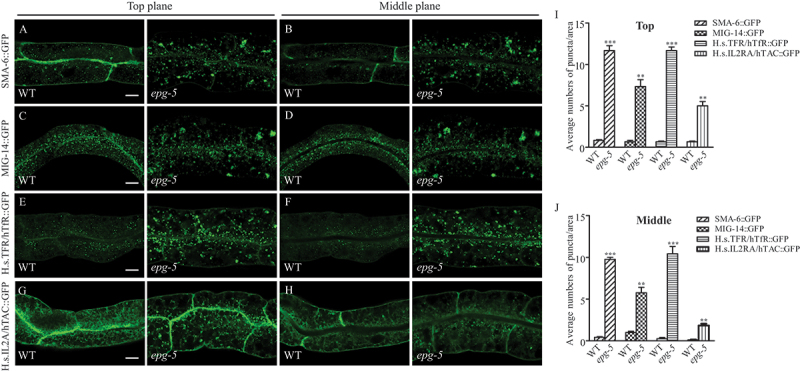
EPG-5 is required for retrograde endocytic trafficking and basolateral endocytic recycling process in the intestine. (A, C, E, G) Localization of SMA-6::GFP, MIG-14::GFP, H.s.TFR/hTfR::GFP, and H.s.IL2RA/hTAC::GFP in in top focal planes of WT and *epg-5* mutant intestine. (B, D, F, H) Localization of SMA-6::GFP, MIG-14::GFP, H.s.TFR/hTfR::GFP, and H.s.IL2RA/hTAC::GFP in the middle focal planes of WT and *epg-5* mutant intestine. WT intestine contains a few small puncta labelled with the indicated markers, while in the *epg-5* mutant intestine, more accumulated puncta are formed. (I-J) Quantification of numbers of puncta (≥1 μm in diameter) labelled with the indicated markers in WT and *epg-5* mutant intestine. Scale bars: 10 μm.

EPG-5 has been shown expressing widely including pharyngeal and body wall muscles, intestinal cells, hypodermal cells [26] and EGL-20/WNT-producing cells (Figure S2W). We next investigated whether loss of *epg-5* activity impairs the retrograde trafficking of components of TGFB and WNT signaling. SMA-6 undergoes retrograde trafficking, in which cargos are delivered from endosomes to the TGN, before they are eventually recycled back to the plasma membrane [14]. SMA-6::GFP, specifically expressed in the intestine, was used to examine retromer-dependent retrograde recycling in intestinal epithelia. In wild-type (WT) animals, SMA-6::GFP mainly localizes on the plasma membrane and only a few intracellular puncta are detected. Loss of function of *epg-5* caused accumulation of much larger SMA-6 puncta in the cytoplasm, which are rare in the WT worms (Figure 2A,B). The number of dispersed SMA-6::GFP puncta was also increased (Figure 2I,J), while the distribution of DAF-4 remained unchanged in *epg-5* mutants (Figure S2U-V).

MIG-14, encoding the *C. elegans* WLS/Wntless homolog, also undergoes retromer-dependent retrograde recycling [25,38]. In the intestinal cells of WT worms, MIG-14::GFP formed some small puncta in the cytoplasm, while in the *epg-5* mutant MIG-14::GFP puncta accumulated in much larger vesicles in the cytosol (Figure 2C,D,I,J). The endocytic trafficking defects, indicated by SMA-6::GFP and MIG-14::GFP, occurred in a temporal-dependent manner, appearing in the first larval (L1) stages (data not shown), becoming most evident in the fourth larval (L4) stage, and being largely attenuated in adult stages (Figure S2A-H, S2X). In summation, these results suggest that EPG-5 is required for retrograde endocytic trafficking of SMA-6 and MIG-14.

### EPG-5 is also required for basolateral endocytic recycling

We then examined the basolateral endocytic recycling process of intestinal cells in *epg-5* mutants. The trafficking of the intestinally expressed H.s.TFR/hTfR and H.s.IL2RA/hTAC have been widely used to examine clathrin-dependent and clathrin-independent endocytosis in the *C. elegans* intestine, respectively. H.s.TFR/hTfR::GFP and H.s.IL2RA/hTAC::GFP are endocytosed into RAB-5-positive early endosomal structures, then are sorted into RAB-10-labeled subdomains of recycling endosomes. Ultimately they are recycled back to the basolateral plasma membrane in a RME-1 (receptor mediated endocytosis 1)-dependent manner [39]. In WT animals, H.s.TFR/hTfR::GFP and H.s.IL2RA/hTAC::GFP are localized to basolateral membranes, with apparent localization to the plasma membrane and proximal small endosomal vesicles and tubules (Figure 2E–H left panels). In *epg-5* mutants, H.s.TFR/hTfR::GFP and H.s.IL2RA/hTAC::GFP accumulated into large aggregates in intestinal cells (Figure 2E–H right panels, 2I-J). These aggregates appeared in L1 stage (data not shown), and became the most evident in L4 stage, then were largely attenuated in adult stages (Figure S2I-P, S2X). We also noted that the ratio of the intensity of H.s.TFR/hTfR::GFP signals on basolateral plasma membrane to those in the cytosol of intestinal cells was dramatically reduced in *epg-5* mutants (Figure 2E,F). In *rme-1* mutants, H.s.TFR/hTfR and H.s.IL2RA/hTAC accumulate in the intestine mainly in adult stages and recycling cargoes decorated the rim of enlarged vacuoles in *rme-1* RNAi worms [40] (Figure S2S-T). We also observed SMA-6::GFP locates on the rim of abnormal vacuoles in these two mutant adult worms (Figure S2Q-R), indicating that RAB-10 and RME-1 are required for the trafficking process of SMA-6. However, *epg-5* mutant intestine accumulates numerous enlarged vesicular structures at larval stages and largely disappear at the adult stage [30]. These enlarged vesicles are distinct from the vacuoles accumulated in *rme-1* and *rab-10* mutants, in which enlarged vacuoles accumulate in the intestine of adult worms and much fewer accumulation in larvae [41,42]. These data collectively suggest that loss of function of *epg-5* impairs basolateral endocytic recycling in the intestine in a manner different from *rme-1* and *rab-10*.

### *Endocytic trafficking cargos are trapped within hybrid endosomal vesicles in* epg-5

**Figure 3.**
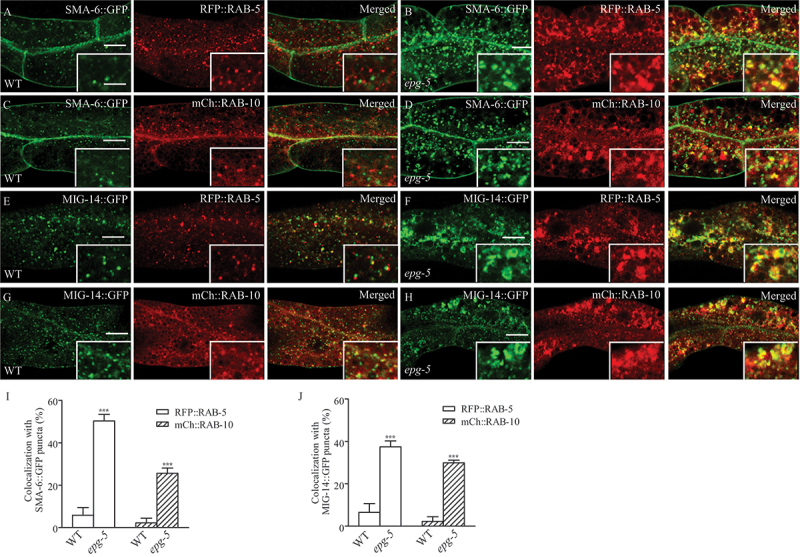
Endocytic trafficking cargos are trapped within hybrid endosomal vesicles in *epg-5* mutants. (A-H) Localization of SMA-6:GFP, MIG-14::GFP, RFP::RAB-5, and mCherry::RAB-10 in WT and *epg-5* mutant intestine. The WT intestine contains some small SMA-6::GFP or MIG-14::GFP puncta that are distinct from RFP::RAB-5 (A,E) or mCherry::RAB-10 (C,G) puncta. In *epg-5* mutant intestine, both SMA-6::GFP and MIG-14::GFP puncta accumulate and partially colocalize with enlarged RFP::RAB-5 (B, F) or mCherry::RAB-10 (D,H) punctate structures. (1, J) Quantification of colocalization of SMA-6::GFP (I) or MIG-14::GFP (J) with indicated markers in WT and *epg-5* mutant intestine (puncta ≥1 μm in diameter were examined on five focal planes in at least three different animals). Scale bars: 10 μm. Inserts: 5 μm.

We next determined where the recycling cargos are trapped in *epg-5* mutants. As mentioned above, *epg-5* mutant intestine accumulates numerous enlarged vesicular structures that are labeled by markers for various endosomal structures, such as RAB-5 (early endosomes), RAB-7 (late endosomes) and RME-1 (recycling endosomes) [30]. The retrograde recycling cargos SMA-6 and MIG-14 were mostly absent from the endosomal vacuoles in the intestine of WT worms (Figure 3A,C–J), but were trapped in abnormal RAB-5- or RAB-10 labeled endosomal structures in the *epg-5* mutant intestine (Figure 3B,D–J). SNX-1, a component of the multisubunit retromer complex that mediates cargo sorting from the early endosome to the Golgi complex [43], formed much larger puncta in the cytosol of *epg-5* mutant intestinal cells, and these SNX-1 puncta colocalized with SMA-6::GFP in *epg-5* mutant intestine (Figure S3A-B, S3I). SNX-3, a non-canonical retromer subunit that is required for the retrograde transport of MIG-14 [17], formed puncta that colocalized with MIG-14::GFP in *epg-5* mutant animals (Figure S3C-D, S3J).

We further used H.s.TFR/hTfR:GFP as an intestinal endocytic trafficking cargo to probe the abnormality of endosomal vesicles in *epg-5* mutants. In the WT intestine, H.s.TFR/hTfR:GFP-positive small puncta are barely colocalized with punctate structures labeled by RFP::RAB-5, or mCherry::RAB-10 in the cytosol of intestinal cells, while in *epg-5* mutants, H.s.TFR/hTfR:GFP formed larger puncta that were colocalized with enlarged structures labeled by RFP::RAB-5, and mCherry::RAB-10 (Figure S3E-H, S3K). These results together indicate that cargos are trapped in hybrid endosomal structures during the endocytic trafficking process in *epg-5* mutants.

### Loss of function of other core autophagy genes fails to cause defective trafficking

**Figure 4.**
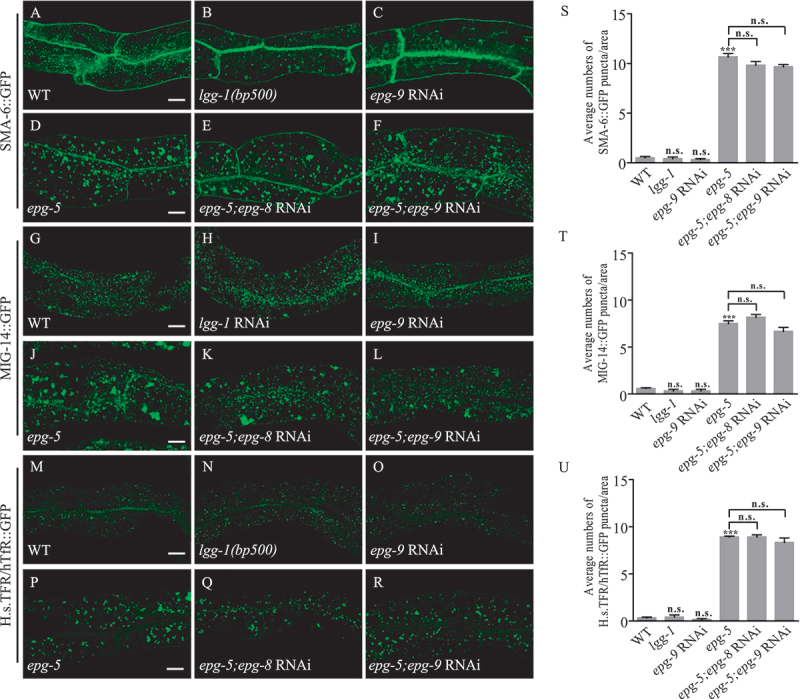
EPG-5 functions in the endocytic trafficking process independent of its role in autophagosome maturation. (A-R) Loss of function of *lgg-1* or *epg-9* could not induce the defective accumulation of SMA-6::GFP (A-C), MIG-14::GFP (G-I), or H.s.TFR/hTfR::GFP (M-O). Loss of function of *epg-8* or *epg-9* could not ameliorate the defective accumulation of SAM-6::GFP (D-F), MIG-14::GFP (J-L), or H.s.TFR/hTfR:GFP (P-R) in the *epg-5* mutant intestine. (S-U) Quantification of numbers of puncta (≥1 μm in diameter) labelled with indicated markers. Scale bars: 10 μm.

We have shown that loss of function of other autophagy genes did not exhibit the short worm body size (Figure 1A), then we want to detect if they affect the endocytic trafficking process as *epg-5* mutants. We found the core autophagy genes such as *lgg-1*, and *epg-9/ATG101* did not affect the endocytic trafficking processes of SMA-6 (Figure 4A–C,S), MIG-14 (Figure 4G–I,T), and H.s.TFR/hTfR (Figure 4M–O,U). When knocking down *epg-8/ATG14* or *epg-9* which both functions upstream of *epg-5* in autophagic pathway by RNAi in *epg-5* mutants, the recycling of these cargoes was still defective, resembling the *epg-5* mutant worms (Figure 4D–F, J-L, P-R, S-U). These results indicate that the defective endocytic trafficking in *epg-5* mutants is not caused by impaired autophagy.

### *Defective RAB conversion on the hybrid vesicles in* epg-5 *mutants*

**Figure 5.**
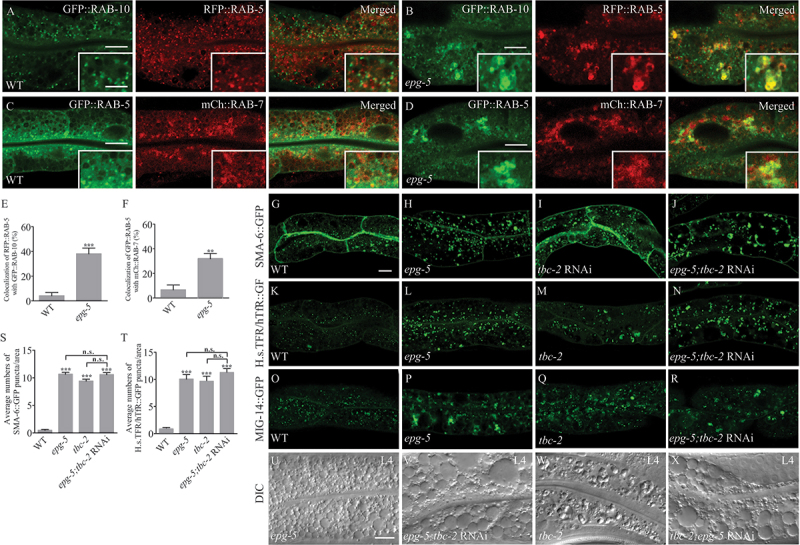
*Epg-5* mutants show defective RAB conversion on the hybrid vesicles. (A-D) Localization of indicated markers in WT and *epg-5* mutant intestine. In WT, RAB-5-, RAB-10-, and RAB-7-positive puncta barely colocalize (A, C), while in the *epg-5* mutant, both enlarged RAB-10-positive (B) and RAB-7-postive structures (D) colocalize with accumulated RAB-5-positive structures. (E-F) Quantification of colocalization of indicated markers in WT and *epg-5* mutant intestine (puncta ≥1 μm in diameter were examined on five focal planes in at least three different animals). (G-R) Localization of SMA-6::GFP, H.s.TFR/hTfR::GFP, and MIG-14::GFP under *tbc-2* depletion. Depletion of TBC-2 induces the accumulation of SMA-6::GFP (I), H.s.TFR/hTfR:GFP (M), and MIG-14::GFP (Q), but does not further exacerbate the accumulation of SMA-6::GFP (J), H.s.TFR/hTfR::GFP (N) or MIG-14::GFP (R) in *epg-5* mutant intestine. (S-T) Quantification of numbers of puncta (≥1 μm in diameter) labelled with indicated markers in WT, *epg-5*, *tbc-2*, and *epg-5; tbc-2* RNAi intestine. (U-X) Differential interference contrast (DIC) images of the L4 larvae intestine of the *epg-5* mutant, *epg-5; tbc-2* RNAi, *tbc-2* mutant, and *tbc-2; epg-5* RNAi. *epg-5* mutant intestine shows abnormal vesicular structures without refractile material inside (U), while *tbc-2* mutant intestine accumulates enlarged vesicles with refractile material inside (W). Loss of function of both *epg-5* and *tbc-2* (V, X) causes vesicular structures without refractile material inside, which resemble those in the *epg-5* mutant intestine. Scale bars: 10 μm. Inserts: 5 μm.

Endocytosed transmembrane proteins that are destined to recycle back to the plasma membrane are sorted into the RAB-10-rich subdomains of early/sorting endosomes, following the recycling process back to the plasma membrane [40,44,45]. The switch of endosome identity is driven by the conversion from RAB-5 to RAB-10, which proceeds with the disassociation of RAB-5 and concomitant acquisition of RAB-10 [46]. In the intestinal cells of WT worms, RAB-5-labeled puncta and RAB-10-positive puncta were barely colocalized (Figure 5A,E). In contrast, we found that the abnormally enlarged RAB-10-positive endosomal structures were partially colocalized with RAB-5 structures in *epg-5* (Figure 5B,E), suggesting that the switch of RAB-5-to-RAB-10 is impaired in *epg-5* mutants. We further performed time-lapse of RFP::RAB-10 and GFP::RAB-5 to catch the transition process in the intestine of WT and *epg-5* mutants. Contrast to the quick contact-and-separation pattern of RAB-5 and RAB-10 in WT worms (Figure S4N), enlarged RAB-5- and RAB-10-positive endosomal puncta sustained much longer in *epg-5* mutants (Figure S4O), confirming the switch defect of RAB-5-to-RAB-10 by the loss of EPG-5.

In a similar manner, the switch of early-to-late endosomes is driven by the conversion from RAB-5 to RAB-7 [47]. In the intestinal cells of WT worms, RAB-5-labeled puncta and RAB-7-positive puncta were largely separable (Figure 5C,E). In *epg-5* mutants, enlarged RAB-5 colocalized with RAB-7 puncta (Figure 5D,F). By time-lapse of RFP::RAB-5 and GFP::RAB-7 to observe the endosomal maturation process, we also found extended colocalization time of enlarged RAB-5- and RAB-7-positive endosomal structure in *epg-5* (Figure S4Q), which was different from the kiss-and-run pattern in WT (Figure S4P), indicating that the endosomal maturation controlled by RAB transition was blocked largely in *epg-5* mutants. All the above results indicate that the enlarged endosomes in *epg-5* mutants are impaired in sorting and maturation of early endosomes, which results in the trapping of endocytosed cargos in RAB-5, RAB-7, or RAB-10-positive endosomal structures.

*C. elegans* TBC-2, which is homologous to human TBC1D2 and TBC1D2B, has been shown to regulate endosome maturation by acting as a RAB-5 GAP to regulate RAB-5, and RAB-7 and RAB-5, and RAB-10 conversion [46,48]. Loss of function of TBC-2 disrupted the retrograde transport of SMA-6 (Figure 5I,S), MIG-14 (Figure 5Q,U), and the basolateral recycling process of H.s.TFR/hTfR (Figure 5M,T). However, depletion of TBC-2 did not further exacerbate the endocytic trafficking defect in *epg-5* mutants (Figure 5J,N,R–T). Distinct from the abnormal vesicular structures found in *epg-5* mutants, refractile material accumulated in the enlarged vesicular structures in the *tbc-2* mutant intestine (Figure 5W). This material was sustained at the larval and adult stages. While the enlarged vesicles in the *tbc-2* mutant intestine were labeled by RAB-7 and LMP-1, they were not labeled by markers for recycling endosomes [48]. In the *epg-5; tbc-2* double mutants, the vacuoles resembled those in the *epg-5* mutant intestine, which had no refractile material inside, although the size of vacuoles were larger than those in *epg-5* mutants at the larval stages (Figure 5U–X). These results indicate that EPG-5 May collaborate with TBC-2 in the endocytic trafficking pathway.

We next determined whether TBC-2 is involved in the autophagy pathway. In *tbc-2* mutants, no obvious accumulation of SQST-1::GFP aggregates was detected in embryonic and larval stages (Figure S4A-C, S4G-I), and depletion of TBC-2 did not enhance the autophagy defect seen by abnormal SQST-1 accumulation in *epg-5* mutants (Figure S4F, S4L, S4M). Thus, *tbc-2* is not essential for autophagy. These results suggest that EPG-5 functions in autophagosome maturation largely independent of TBC-2.

We then detected if the localization of TBC-2 would change in *epg-5* mutant background. We found the normal endosomal distribution of mCherry::TBC-2 in the intestine of WT worms (Figure S5A) [46]. In contrast, mCherry::TBC-2 formed big vesicle-structures in the absence of EPG-5 (Figure S5B), indicating that EPG-5 is important for the normal endosomal distribution of TBC-2. We further found these big vesicle-structures in *epg-5* mutants were VPS-18 positive. In WT worms, mCherry::TBC-2 puncta located apart from or close to VPS-18-positive endolysosomes (Figure S5C-E). However, big vesicle-structures labeled by mCherry::TBC-2 in the intestine of *epg-5* mutants were mostly colocalized with GFP::VPS-18 (Figure S5F-H), suggesting the incorrect membrane localization of TBC-2 in the absence of EPG-5. By GST-affinity isolation approach, we also found TBC-2 can interact with 375-696aa of EPG-5 (Figure S5O), which has been shown to interact with assembled SNARE motifs of SYX-17/STX-17-SNAP-29-VAMP-7 complex [30]. These results indicate that EPG-5 is important for TBC-2 function.

### *The trafficking defect in* epg-5 *mutants is suppressed by facilitated RAB conversion*

**Figure 6.**
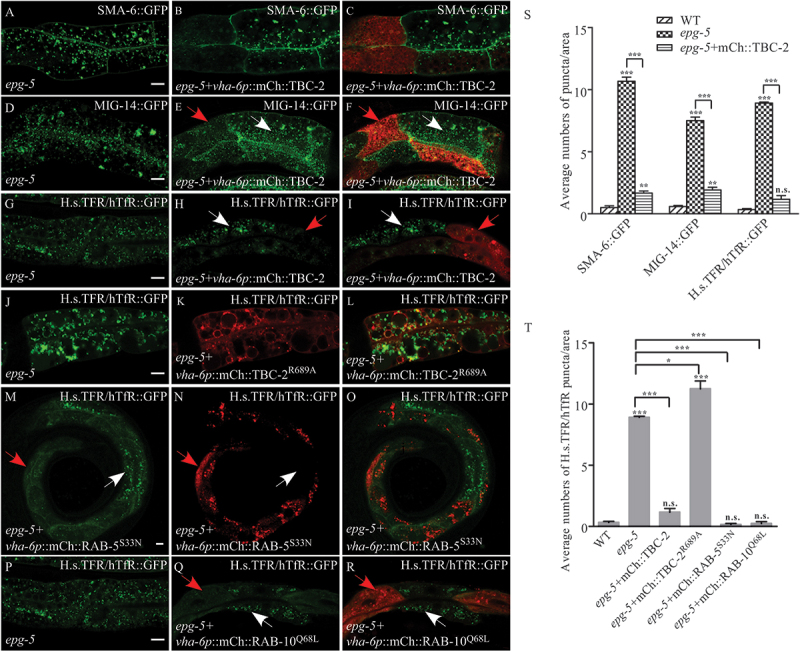
Facilitated RAB conversion could suppress the endocytic trafficking defect in *epg-5* mutants. (A-I) The *tbc-2* transgene expressed in the intestinal cells could rescue the endocytic trafficking defect of cargos SMA-6 (A-C), MIG-14 (D-F), and H.s.TFR/hTfR (G-I) in the *epg-5* mutant intestine. Red arrows indicate the areas with expression of *tbc-2* transgene, where the endocytic defect of cargos is rescued. White arrows indicate the areas without visible expression of *tbc-2* transgene, where the cargos are defectively accumulated. (J-L) Overexpression of TBC-2^R689A^ with impaired GAP activity could not rescue the endocytic trafficking defect of H.S.TFR/hTfR in the *epg-5* mutant intestine. (M-R) Overexpression of the dominant-negative RAB-5^S33N^ (M-O) and Constitutively activated RAB-10^Q68L^ (P-R) could largely rescue the endocytic trafficking defect of H.s.TFR/hTfR in the *epg-5* mutant intestine. Red arrows indicate the areas with expression of transgene, where the endocytic defect of cargos is rescued. White arrows indicate areas without visible expression of transgene, where the cargos H.s.TFR/hTfR are defectively accumulated. (S) Quantification of numbers of puncta (≥1 μm in diameter) labelled with the indicated markers in the intestine of WT, *epg-5* mutants, and *epg-5* mutants with TBC-2 overexpression. (T) Quantification of numbers of H.s.TFR/hTfR puncta (≥1 μm in diameter) in WT, *epg-5* mutants, *epg-5* mutants with overexpression of TBC-2, TBC-2^R689A^, RAB-5^S33N^ or RAB-10^Q68L^. Scale bars: 10 μm.

Since TBC-2 acts as a RAB-5 GAP to regulate RAB-5, and RAB-7 and RAB-5, and RAB-10 conversion, we then examined the effect of TBC-2 overexpression on the recycling defect in *epg-5* mutants. The expression of transgenes from extrachromosomal arrays in *C. elegans* can be mosaic, as it is lost in some cells during development. In the intestinal cells that expressed *tbc-2*, the endocytic recycling defect of cargos SMA-6, MIG-14, and H.s.TFR/hTfR in *epg-5* mutants was rescued (Figure 6A–I,S), while overexpression of a mutant form of TBC-2^R689A^ with impaired GAP activity showed no rescue (Figure 6J–L,T). These results suggest that the GAP activity of TBC-2 is required for the role of EPG-5 in endocytic trafficking.

TBC-2 acts as a GAP for RAB-5 to inactive RAB-5 during endosome maturation [48]. We then determined whether expression of dominant-negative RAB-5 (GDP form), which may facilitate the normal RAB-5, and RAB-10 conversion, affects the endocytic trafficking defect in *epg-5* mutants. Expression of RAB-5^S33N^ (GDP from) largely rescued the endocytic trafficking defect in the *epg-5* mutant intestine (Figure 6M–O,T). Similarly, expression of activated RAB-10^Q68L^ (GTP form) also rescued the endocytic trafficking defect in the *epg-5* mutant intestine (Figure 6P–R,T). Overexpression of dominantly negative RAB-10 (RAB-10^T23N^, GDP form) showed a much less extent rescue (Figure S5I-N), which was probably due to a feedback regulation of RAB-10 dynamics, causing a minor elevation of RAB-10 GTP forms. The active GTP-bound RABs associate with specific membrane domains, allowing them to interact with effector proteins that regulate processes like vesicle budding, tethering, fusion, and transport along the cytoskeleton. In contrast, when in their inactive GDP-bound state, RABs remain sequestered in the cytoplasm [48]. Since EPG-5 contributes to the function of TBC-2, we expected to observe elevated level of membrane-bound RAB-5 caused by abnormally increased level of GTP-bound RAB-5 in *epg-5* mutants. We therefore extended to measure RAB-5 membrane association by separation of membranes from cytosol in worm lysates of WT and *epg-5* mutants and found an elevation of GFP::RAB-5 membrane-to-cytosol ratio in *epg-5* mutants (Figure S5P). These results indicate that EPG-5 acts with TBC-2 in the endocytic trafficking via regulating the RAB dynamics.

### *The HOPS complex is required for the endocytic trafficking defect in the* epg-5 *mutant intestine*

**Figure 7.**
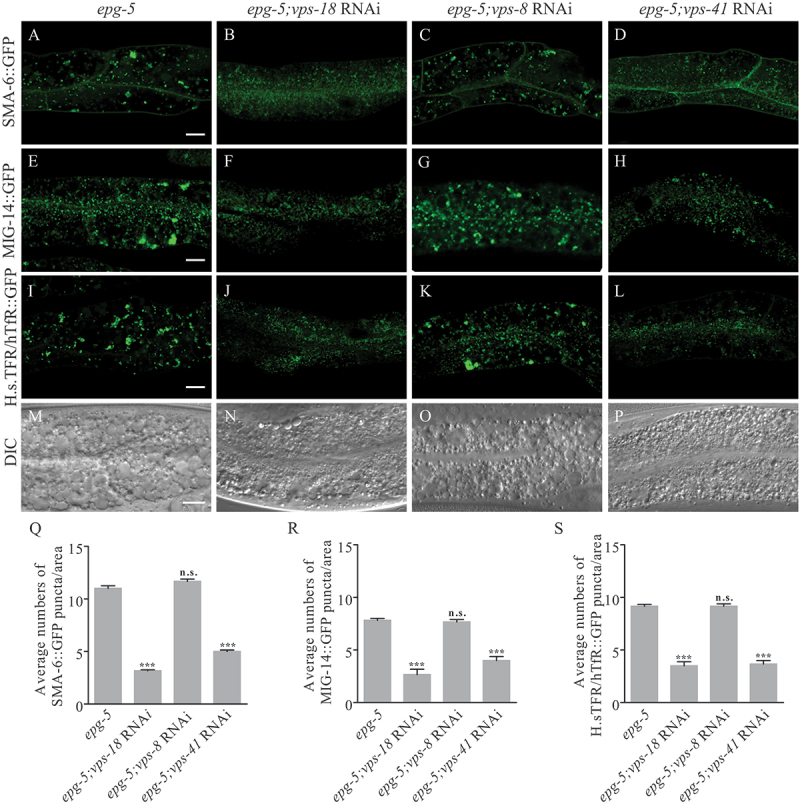
Knockdown of CORVET or HOPS complexes could suppress the endocytic trafficking defect in *epg-5* mutants. (A-L) Loss of function of *vps-18* or *vps-41*, but not *vps-8* could suppress the endocytic trafficking defect of the cargos SMA-6 (A-D), MIG-14 (E-H), and H.s.TFR/hTfR (I-L) in the *epg-5* mutant intestine. (M-P) DIC images show that *vps-18* RNAi or *vps-41* RNAi, but not *vps-8* RNAi could suppress the formation of vacuoles in the *epg-5* mutant intestine. (Q-S) Quantification of numbers of puncta (≥1 μm in diameter) labelled with indicated markers. Scale bars: 10 μm.

The class C core vacuole/endosome tethering (CORVET) and homotypic fusion and vacuole protein sorting (HOPS) complexes are tethering factors within the endolysosomal trafficking system. CORVET and HOPS complexes share 4 core subunits (VPS11, VPS16, VPS18, and VPS33) and possess specific subunits (VPS3 and VPS8 in CORVET, VPS39 and VPS41 in HOPS), which determine their specific binding with RAB5 GTPase and RAB7 GTPase, respectively [49]. We determined the role of these tethering factors in mediating the formation of abnormal vesicles in *epg-5* mutants. *vps-18* RNAi and *vps-41* RNAi, but not *vps-8* RNAi, suppressed the accumulation of endocytic recycling cargoes SMA-6::GFP, MIG-14::GFP, and H.s.TFR/hTfR::GFP in *epg-5* mutant intestine (Figure 7A–L). RNAi inactivation of *vps-18* and *vps-41* also suppressed the formation of vacuoles in the *epg-5* mutant intestine (Figure 7M–P). As shown previously, HOPS is required for the large endosomal structure in *tbc-2* mutants [48] (Figure S6A-A’’). In worm intestinal cells, loss-of P4-ATPase TAT-1 also accumulates large vacuoles of mixed endolysosomal identity [50]. Similarly, *vps-18* RNAi and *vps-41* RNAi suppressed the vacuoles formation in *tat-1* mutants in which endocytic sorting of cargos were also severely affected and showed defective vacuolation in the intestinal cells (Figure S6B-B’’), while they did not affect the accumulation of vacuoles in *rab-10* or *rme-1* mutants (Figure S6C-D’’). We further determined if knockdown of VPS-18 or VPS-41 would also suppress the abnormality of endocytic trafficking in *epg-5* mutants. The intestinal cargo H.s.TFR/hTfR::GFP was no longer trapped in enlarged endosomal structures labeled by RFP::RAB-5 by RNAi inactivation of *vps-18* and *vps-41* in *epg-5* mutants (Figure S6E-G). RAB-5-labeled puncta and RAB-7-positive puncta exhibited largely separable pattern as in WT worms after *vps-18* and *vps-41* RNAi in *epg-5* mutants (Figure S6H-J). These results suggest that the HOPS complex is required for the endocytic trafficking defect in *epg-5* mutant intestine.

## Discussion

The early/sorting endosomes serve as the central hub for endocytic trafficking that transport the endocytosed cargos to different destinations, including to lysosomes for degradation via late endosomes/multivesicular bodies (MVBs), to the plasma membrane via recycling endosomes, or to the Golgi network in a retromer-dependent manner. Here, we demonstrated the participation of EPG-5 in the endocytic trafficking process by modulating endomembrane trafficking. EPG-5 May regulate TGFB and WNT signaling. In *epg-5* mutants, the retrograde endocytic trafficking of TGFB receptor SMA-6 and WNT-binding protein MIG-14/Wntless is impaired (Figure 2). Consequently, the body size is shortened and migration of Q neuroblast descendants along the A/P body axis is defective, processes controlled by TGFB and WNT signaling, respectively (Figure 1). EPG-5 is also required for the basolateral recycling process of H.s.TFR/hTfR and H.s.IL2RA/hTAC in the intestinal cells (Figure 2). SMA-6, MIG-14/Wntless, and H.s.TFR/hTfR were all trapped within hybrid endocytic vesicles with mixed identities, including early endosomes (RAB-5), recycling endosomes (RAB-10), and retromer-contained retrograde trafficking vesicular structures (SNX-1 or SNX-3) (Figure 3). We found that RAB-5, and RAB-10 conversion, which is essential for the sorting of cargos from early endosomes to recycling endosomes, was defective in *epg-5* mutants, and we facilitated this conversion through overexpressing the dominant-negative RAB-5 (GDP form) and activated RAB-10 (GTP form), with both rescuing the endocytic trafficking defect (Figure 5). Consistently, overexpression of TBC-2, which acts as RAB-5 GAP to regulate RAB-5, and RAB-10 conversion in endosome maturation, also largely rescued the endocytic trafficking defect. Moreover, overexpression of TBC-2^R689A^ with defective GAP activity shows no rescue (Figure 6). In addition, we found EPG-5 contributes to the endosomal recruitment of TBC-2. Loss of *epg-5* resulted in enlarged TBC-2 puncta and abnormal distribution of TBC-2 in the intestine (Figure S5). Therefore, EPG-5 acts with TBC-2 to regulate the RAB dynamics, and the GAP activity of TBC-2 is required for the role of EPG-5 in the endocytic recycling process. HOPS complex shows important function in endocytic trafficking by determining the specific binding with RAB-7. Knock-down of either of the 2 subunits of HOPS complex, VPS-18 and VPS-41, could suppress the abnormal accumulation of endocytic recycling cargoes (SMA-6::GFP, MIG-14::GFP, and H.s.TFR/hTfR::GFP), and formation of big vacuoles in the *epg-5* mutant intestine (Figure 7). Also as expected, after inactivation of *vps-18* or *vps-41* by RNAi in *epg-5* mutants, both H.s.TFR/hTfR::GFP and GFP::RAB-7 separates from RFP::RAB-5, indicating the HOPS complex is required for the endocytic trafficking defect in *epg-5* (Figure S6K).

The precise delivery of signal molecules and their receptors to membrane-bound compartments are tightly regulated. The early endosomes serve as the central hub for endocytic trafficking that transport the endocytosed cargo to different destinations. Cargo can be routed to lysosomes for degradation via late endosomes/MVBs, to plasma membrane via recycling endosomes, or to TGN in a retromer-dependent manner. In *epg-5* mutants, type-I receptor SMA-6 in TGFB signaling and WNT-binding protein MIG-14/Wntless, which are both undergo retromer-dependent retrograde trafficking, were trapped in hybrid vesicular structures with the identities of early endosomes (RAB-5) and recycling endosomes (RAB-10), implying that the sorting on early endosomes is disrupted. The type-II receptor DAF-4 in TGFB signaling employs a distinct recycling pathway that is mediated by ARF-6, and its trafficking process is normal in *epg-5* mutants (Figure S1, S2). Thus, EPG-5 is selectively involved in the retromer-dependent retrograde trafficking processes. The E2 ubiquitin-conjugating enzyme UBC-13, which functions in cargo ubiquitination to maintain distinct microdomains on endosomes, is also essential for retrograde transport of MIG-14 and SMA-6. Thus, loss of *ubc-13* results in MIG-14 missorting to late endosomes and lysosomes, but it does not affect recycling of retromer-independent cargoes including H.s.TFR/hTfR:GFP and H.s.IL2RA/hTAC:GFP [51], which is different from *epg-5* mutants. In *epg-5* mutants, both retromer-dependent (SMA-6::GFP, MIG-14::GFP) and retromer-independent (H.s.TFR/hTfR::GFP, H.s.IL2RA/hTAC::GFP) endocytic trafficking cargos are trapped within hybrid endosomal structures because of the defective RAB-5 and RAB-7, and RAB-5 and RAB-10 conversion.

In *C. elegans*, morphogenesis of the male tail is also controlled by SMA and MAB TGFB signaling. However, *epg-5* mutants showed normal development of male-specific sensory rays (Figure S1). Meanwhile, *epg-5* mutants showed no obvious defect in the morphology of six mechanosensory neurons regulated by WNT signals, including the A/P axis polarity of ALM and PLM, axon growth of AVM and PVM, and branch formation of ALM and AVM (data not shown). The selective physiological processes affected in *epg-5* mutants could be due to the spatial expression of EPG-5, the redundancies of regulating factors, or their different sensitivity to the impaired signaling.

Autophagy pathway has multiple intersection points with endocytic trafficking process, such as the fusion of autophagosome with endolysosomal compartment which is essential for autophagosome maturation. Participation of EPG-5 in autophagosome maturation, which is primarily mediated by the binding of EPG-5 with LGG-1 and RAB-7 [30], is not sufficient to induce the endocytic trafficking defect in *epg-5* mutants. Loss of function of autophagy essential genes (*lgg-1, epg-9*) acting upstream of *epg-5* did not cause obvious endocytic trafficking defect, and further depletion of autophagy genes did not exacerbate the endocytic trafficking defect in *epg-5* mutants (Figure 4). EPG-5 seems possessing distinct roles in endocytic trafficking independent of its function in autophagy. Autophagy defects, labeled by GFP::LGG-1 and SQST-1 ectopic accumulation, in *epg-5* mutants persist during the entire larval and adult stages [26]. While the endocytic trafficking defects occur in a temporal-dependent manner, appearing in L1 stage, becoming the most evident in L4 stage and largely attenuated in young adult stage (Figure S2). Previous report shows endocytic degradation and endocytic recycling delays in *Epg5*-deficient cells by *in vitro* [29]. Here, employing *in vivo* model system of *C. elegans*, we further confirmed the participation of EPG-5 in endocytic trafficking process, largely independent of its role in autophagosome maturation.

Recessive mutations in EPG5 cause the multisystem disorder Vici syndrome in humans [27]. *Epg5*-deficient mice display some features of Vici syndrome, including corpus callosum changes and myopathy [29]. *Epg5*-deficient mice also exhibit selective damage of cortical layer 5 pyramidal neurons and spinal cord motor neurons, the key manifestations of amyotrophic lateral sclerosis (ALS) [28]. Other studies have shown that impairment of the endosomal sorting complex caused accumulation of non-degradative autophagic vacuoles, a result has been linked with ALS [52–55]. Our study here suggests that both accumulation of non-degradative autophagic vacuoles and defective endocytic trafficking contribute to the pathogenesis associated with *EPG5/EPG-5* depletion.

## Materials and methods

### Worm strains and maintenance

Strains used in this study were listed here:

Bristol N2 strain was used as WT. *epg-5(bp450)*, *epg-5(tm3425)*, *epg-1(bp414)*, *epg-6(bp242)*, *atg-18(gk378)*, *vps-29(tm1320)*, *lgg-1(bp500)*, *tbc-2(tm2241)*, *tat-1(qx30)*, *rab-10(dx2)*, *rme-1(b1045)*, *bpEx340(epg-5p::epg-5::GFP, unc-76)*, *pwIs921(vha-6p::sma-6::GFP, unc-76)*, *pwIs922(vha-6p::daf-4::GFP, unc-76)*, *cgEx1(vha-6p::epg-5::GFP, unc-76)*, *cgEx2(semo-1p::sma-6::mCherry, unc-76)*, *cgEx3(epg-5p::GFP)*, *zdIs5(mec-4p::GFP, unc-76)*; *casIs35(gcy-32p::mCherry, unc-76)*, *pwIs765(vha-6p::mig-14::GFP, unc-76)*, *pwIs717(vha-6p::H.s.TFR/hTfR::GFP, unc-76)*, *pwIs112(vha-6p::H.s.IL2RA/hTAC::GFP, unc-76)*, *pwIs846(vha-6p::RFP::rab-5, unc-76)*, *pwIs414(vha-6p::mCherry::rab-10, unc-76)*, *qxIs111(ges-1p::mCherry::rab-7, unc-76)*, *qxIs195(ges-1p::GFP::rab-10, unc-76)*, *qxEx2841(vha-6p::GFP::rab-5, unc-76)*, *bpEx338(vha-6p::snx-1::mCherry, unc-76)*, *bpEx339(vha-6p::snx-3::mCherry, unc-76)*, *bpIs151(sqst-1p::sqst-1::GFP, unc-76)*, *bpIs267(semo-1p::sqst-1::GFP, unc-76)*, *vhIs1(vha-6p::mCherry::tbc-2, unc-76)*, *pwIs921(vha-6p::sma-6::GFP, unc-76); [vha-6p::mCherry::tbc-2 + ord-1p:RFP], pwIs765(vha-6p::mig-14::GFP, unc-76); [vha-6p::mCherry::tbc-2 + ord1p::RFP]*, *pwIs717(vha-6p::H.s.TFR/hTfR::GFP, unc-76); [vha-6p::mCherry::tbc-2 + ord-1p::RFP], pwIs717(vha-6p::H.s.TFR/hTfR::GFP, unc-76); [vha-6p::mCherry::tbc-2 r689a + ord-1p::RFP], pwIs717(vha-6p::H.s.TFR/hTfR::GFP, unc-76); [vha-6p::mCherry::rab-5 s33n + ord-1p::RFP], pwIs717(vha-6p::H.s.TFR/hTfR::GFP, unc-76); [vha-6p::mCherry::rab-10 q68l + ord-1p::RFP]*, *pwIs717(vha-6p::H.s.TFR/hTfR::GFP, unc-76); [vha-6p::mCherry::rab-10 t23n + ord-1p::RFP], yqIs26(vps-18p::vps-18::GFP)*, *bxIs16(cat-2::YFP + tph-1::CFP)*. All *epg-5* mutants without labeling are *tm3425* alleles. Animals carrying the integrated or transgenic arrays were outcrossed with the N2 strain three times. All worm strains were grown at 20°C on standard nematode growth media (NGM: 20 g/L agar [Sigma, A1296], 2.5 g/L peptone [Sigma, P5905], 3 g/L NaCl, 1 mM CaCl_2_, 1 mM MgSO_4_, 25 mM K_3_PO_4_, 13 μM cholesterol [Sigma, C8667]) plates seeded with *E. coli* strain OP50.

### RNAi inactivation experiments

For RNAi injection experiments, dsRNAs were synthesized as described previously and injected into worms [30].

Primers for *epg-5* dsRNA synthesis are: 5’-CACTAGTAATACGACTCACTATAGGGGACAACATTCGAACGTCTTC-3’ and 5’-CACTAGATTTAGGTGACACTATAGAAGATCATCAATTAGACGTCGG-3’. Primers for *epg-8* dsRNA synthesis are: 5’-CACTAGTAATACGACTCACTATAGGGAACTACGAGCAAAGCTAGC-3’ and 5’-CACTAGATTTAGGTGACACTATAGAAGTCCAAGATTCATCGAGTTCG-3’. Primers for *epg-9* dsRNA synthesis are: 5’-CACTAGTAATACGACTCACTATAGGGAGTGCTGGATATTTGCGAGC-3’ and 5’-CACTAGATTTAGGTGACACTATAGAAGAAGACGAATGAGTCTCGAC-3’. Primers for *tbc-2* dsRNA synthesis are: 5’-CACTAGTAATACGACTCACTATAGGGCGACGAGTTCGTGATCTTGA-3’ and 5’-CACTAGATTTAGGTGACACTATAGAAGTTCTTGCCGACTCTTCGAG-3’. Primers for *vps-8* dsRNA synthesis are: 5’-CACTAGTAATACGACTCACTATAGGGTGCTACCGTAATCCGACAAC-3’ and 5’-CACTAGATTTAGGTGACACTATAGAAATGAGAGCTCCATCGAGAAC-3’. Primers for *vps-18* dsRNA synthesis are: 5’-CACTAGTAATACGACTCACTATAGGGATGTCACTTCCTCTCAACGG-3’ and 5’-CACTAGATTTAGGTGACACTATAGAATAAGCCTGCTTGAAGAGACG-3’. Primers for *vps-41* dsRNA synthesis are: 5’-CACTAGTAATACGACTCACTATAGGGATGGCACTCCGACATTGGATC-3’ and 5’-CACTAGATTTAGGTGACACTATAGAATCAGCAAACTCAGTTCCCTCG-3’. Feeding RNAi was performed using *E. coli* HT115 carrying the L4440 plasmid as empty vector control or double-stranded RNA-expressing plasmids targeting the genes of interest. RNAi bacteria were cultured overnight at 37°C and then seeded onto the NGM plates supplemented with 100 μg/mL ampicillin (Beyotime, ST0007) and 1 mM isopropyl β-D-thiogalactopyranoside (IPTG, Sigma, I5502). The progeny was examined for the corresponding phenotype.

### Body-length measurement

Live hermaphrodite worms were selected at late L4 stage on 2% agarose pads, treated with levamisole and photographed using a microscope with a digital camera system (Zeiss Corporation). Lengths of worms were measured in ImageJ software using segmented lines.

### *Quantification of the migration of Q* cell descendants

The migration of PQR and PVM neurons, which could be respectively tracked by *gcy-32p*::mCherry and *mec-4p*::GFP, were measured according to their final positions relative to nonmotile neurons URX and PLM. The relative position of PQR (or PVM) was calculated as the distance between URX and PQR (or PVM) divided by the distance between URX and PLM, as shown in the schematic diagram in Figure S1D. Distances were measured using ImageJ Software. We quantified the percentages of neuron cells at each position relative to URX or PLM neurons using Microsoft Excel Software and made the distribution chart of final positions of PQR and PVM neurons in GraphPad Prism 5 Software.

### *Endocytic recycling in* C. elegans

Numbers of SMA-6:GFP, MIG-14::GFP, H.s.IL2RA/hTAC::GFP and H.s.TFR/hTfR::GFP puncta (≥1 μm in diameter) in the cytoplasm in intestinal cells were determined within a unit area of 1,000 μm^2^ using ImageJ. At least twelve different areas from 3–5 L4 larvae (≥4 areas for each animal) were quantified for each genotype.

### Live worm confocal microscopy

Live hermaphrodite worms were selected from 2% agarose pads, treated with levamisole, and multiwavelength fluorescence images were captured using a Zeiss LSM 710 Meta microscope. For time-lapse fluorescence, images were captured every 30 s for 5 min then processed and viewed using ZEN 3.1 software. Quantification of puncta numbers and colocalization analysis were performed with ImageJ Software. For colocalization, the numbers of puncta (≥1 μm in diameter) and colocalized puncta (more than 50% overlapping area) were determined, respectively. Then we calculated the percentage of colocalization. At least twelve different areas from 3–5 L4 larvae (≥4 areas for each animal) were quantified for each genotype.

### In vitro *affinity-isolation assay*

MBP-His_6_-tag fused EPG-5 (375-696aa) was expressed and purified as previously described [30]. Full-length TBC-2 was cloned into the pGEX-6P-1 vector (NovoPro, V010912) to produce GST-tagged proteins. Soluble recombinant proteins were expressed in *E. coli* BL21-CodonPlus (DE3) and purified on glutathione Sepharose 4B beads (for TBC-2; Cytiva, 17075601) and Ni-NTA agarose beads (for EPG-5; QIAGEN, 30210). GST or GST-tagged TBC-2 (20–30 μg) was incubated with GST beads in 500 μL affinity-isolation buffer (1×PBS [Sigma, P3813] plus 1‰ Triton X-100 [Sigma, P8787]) for 30 min at 4°C following incubation with 20 μg MBP-tagged EPG-5 in 500 μL affinity-isolation buffer for 1 h at 4°C. The beads were washed 3–4 times with the affinity-isolation buffer, and the bound proteins were boiled in 5×SDS sample loading buffer, separated by SDS-PAGE and visualized by immunoblotting of anti-MBP (1:2,000; Sigma, M6295).

### Worm membrane fractionation and western analysis

Worms expressing intestinal GFP::RAB-5 in WT and *epg-5* mutants were synchronized and cultured on NGM plates. L4 stage worms were washed off with M9 buffer (3 g/L KH_2_PO_4_, 6 g/L Na_2_HPO_4_, 5 g/L NaCl, 1 mM MgSO_4_), pelleted and resuspended in lysis buffer (50 mM Tris-HCl, pH 8.0 [Sangon Biotech, A100234], 2 mM DL-dithiothreitol [DTT, MedChemExpress, HY-15917], 20% Sucrose [Sigma, V900116], 10% Glycerol [Sigma, G5516], protease inhibitor cocktail [Sigma, P2714]) and performed membrane fractionation as previously described [46]. Basically, the worms were first disrupted using a FastPrep-24 homogenizer (MP Biomedicals) following centrifugation at 1,000 g for 5 min at 4°C. Then the post-nuclear lysate was centrifuged at 100,000 g for 1 h to separate the pellet (membrane part) and supernatant (cytosol part). Both the pellet and supernatant were subjected to SDS-PAGE and GFP were detected using corresponding primary (anti-GFP, 1:1,000; Proteintech, PABG1–100) and HRP-conjugated secondary antibody (anti-ACTB, 1:5,000; Proteintech 66009–1-Ig).

### Statistical analyses

All statistical analyses were performed in GraphPad prism 5.0. Two-tailed unpaired *t*-test was used for statistical analysis of two groups of samples, one-way or two-way ANOVA was used for statistical analysis of more than two groups of samples. Data were presented as mean ± SEM. *p* < 0.05 was considered a significant difference, * represents *p* < 0.05, ** represents *p* < 0.01, *** represents *p*< 0.001, **** represents *p* < 0.0001, n.s. represents no significant difference.

## Supplementary Material

worm membrane trafficking supp figs_final 03202025.docx
