# Supplementary material for: EPG-5 regulates TGFB/TGF-β and WNT signalling by modulating retrograde endocytic trafficking
Source: Autophagy. 2025 Apr 3;21(9):1995–2008. doi: 10.1080/15548627.2025.2485420 (PMC12363523; doi:10.1080/15548627.2025.2485420)
Supplement: worm membrane trafficking supp figs_final 03202025.docx [file KAUP_A_2485420_SM1215.docx]

**
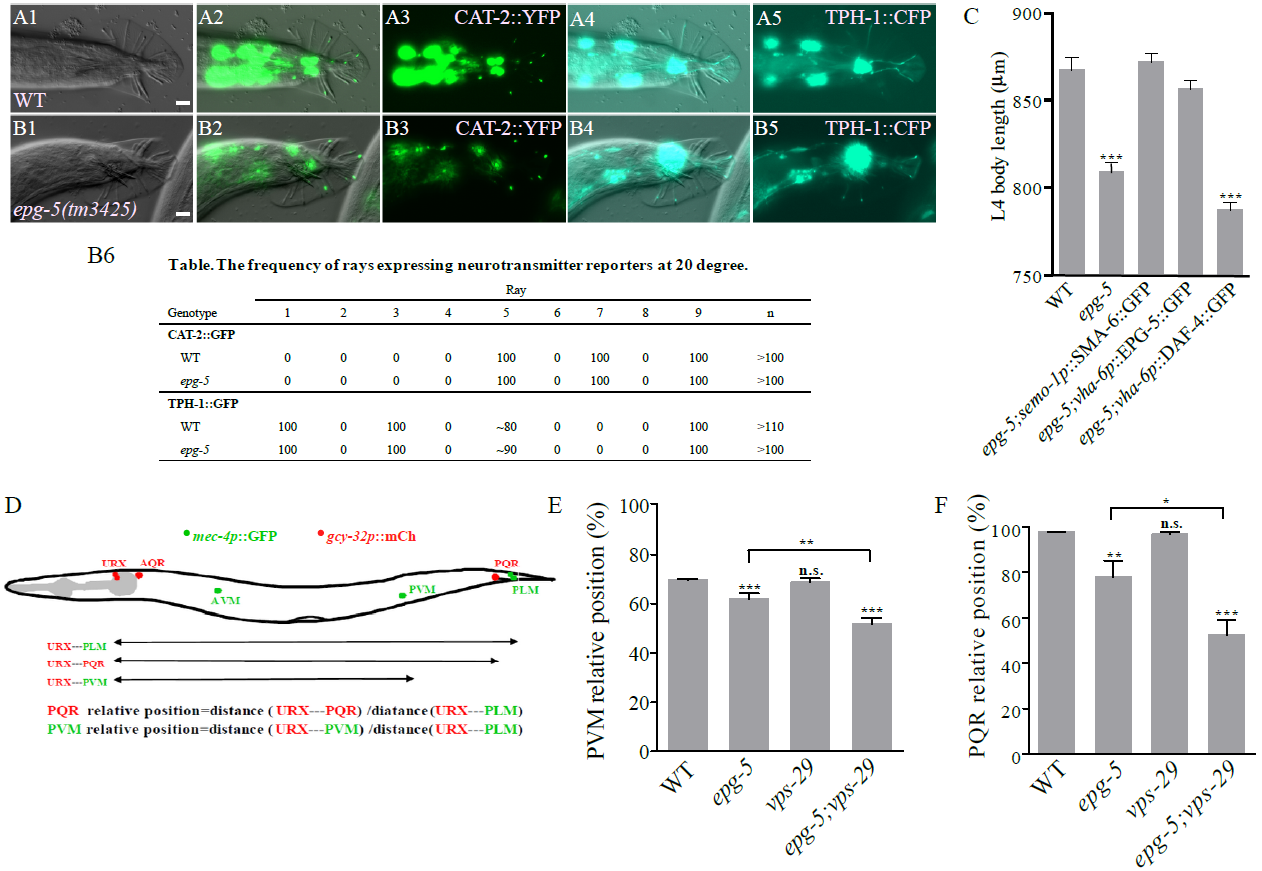
**

**Figure S1.** Phenotypes associated with TGFB and WNT signaling in *epg-5* mutants, related to Figure 1. (**A1-B6**) Morphogenesis of the male tail indicated by dopaminergic (CAT-2::YFP; A1-A3, B1-B3, B6) and serotonergic (TPH-1::CFP; A4-A5, B4-B5, B6) ray neurons is normal in *epg-5* mutants. (**C**) Quantification of body length of L4 larvae (n≥ 10) in different genetic backgrounds. Transgenesis using EPG-5::GFP driven by intestine-specific promoter (*vha-6p*) and SMA-6::GFP driven by hypodermis-specific promoter (*semo-1p*) could rescue the defective body size in *epg-5(tm3425)* mutants. Transgenesis using *vha-6p*::DAF-4::GFP could not rescue the defective body size in *epg-5(tm3425)* mutants. (**D**) Schematic strategy for measuring PQR and PVM relative positions. (**E-F**) Quantification of PVM (E) and PQR (F) relative position in WT, *epg-5(tm3425)* mutants, *vps-29(tm1320)* mutants, and *epg-5(tm3425); vps-29(tm1320)* mutants (n > 30), which were determined according to the strategy in Figure S1-D. Scale bars: 10 μm.

**
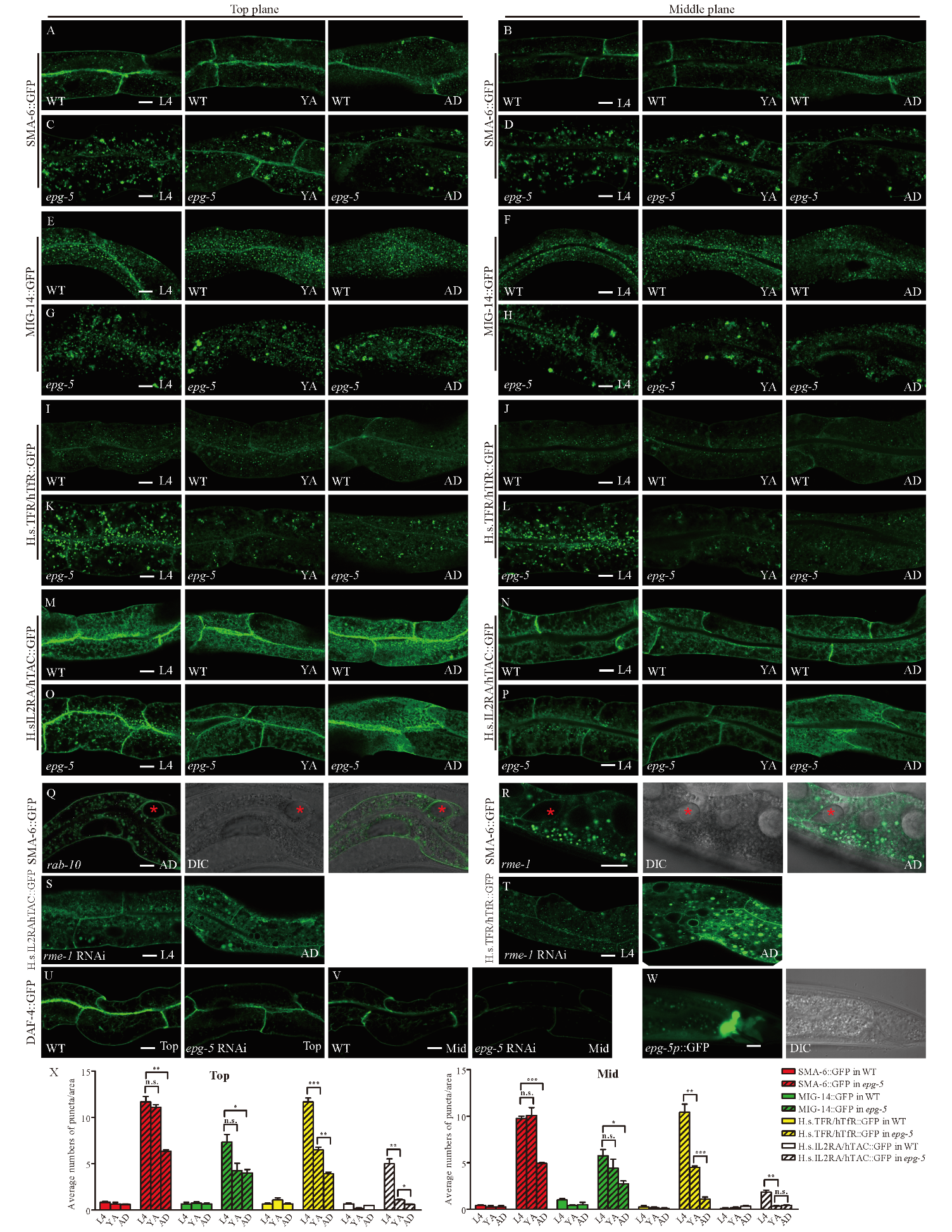
**

**Figure S2.** The endocytic trafficking defects in *epg-5* mutants occur in a temporal-dependent manner, related to Figure 2. ***(*A-D**) Localization of SMA-6::GFP in the intestine of WT and *epg-5* mutant worms in different stages (L4 fourth larvae, YA young adult, AD adult). (**E-H**) Localization of MIG-14::GFP in WT and *epg-5* mutant intestine in different stages. (**I-L**) Localization of H.s.TFR/hTfR::GFP in WT and *epg-5* mutant intestine in different stages. (**M-P**) Localization of H.s.IL2RA/hTAC::GFP in WT and *epg-5* mutant intestine in different stages. (**Q-R**) Localization of SMA-6::GFP in *rab-10* and *rme-1* intestine in adult stage. Red asterisks: enlarged vacuoles. (**S**) Localization of H.s.IL2RA/hTAC::GFP in L4 and adult stages of *rme-1* RNAi worms. (**T**) Localization of H.s.TFR/hTfR::GFP in L4 and adult stages of *rme-1* RNAi worms. (**U-V**) Localization of DAF-4::GFP in the intestine of *epg-5* mutant worms. There is no defective accumulation of DAF-4::GFP. (**W**) DIC and fluorescence images of WT expressing GFP driven by the *epg-5* promoter showing EGL-20 producing cells. (**X**) Quantification of puncta ( ≥ 1μm in diameter) labelled with indicated markers in WT and *epg-5* mutant intestine with different stages. Scale bars: 10 μm.


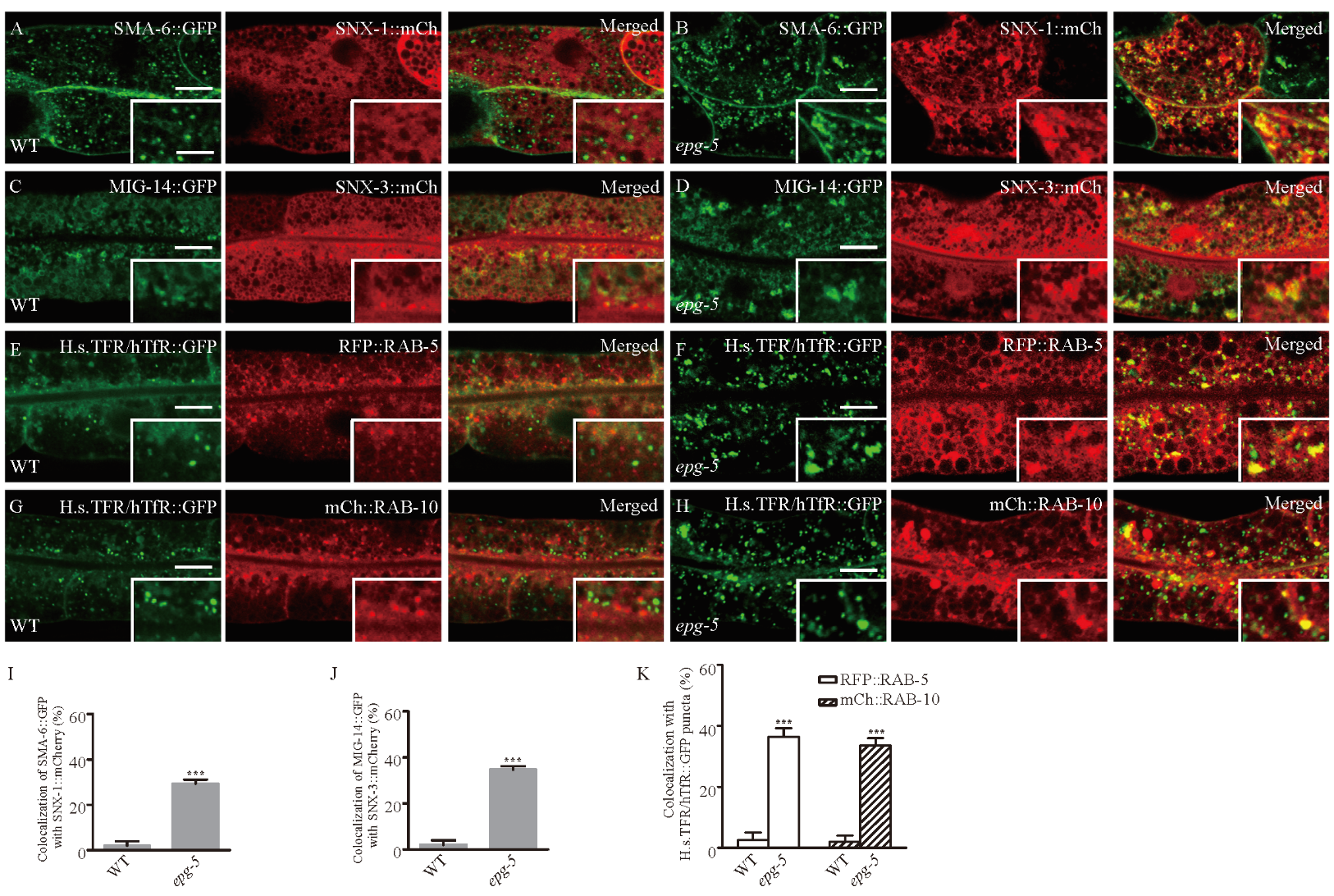


**Figure S3.** *epg-5* mutants exhibit hybrid endosomal vesicles containing endocytic trafficking cargos, related to Figure 3. (**A-B**) Localization of SMA-6::GFP and SNX-1::mCherry in WT and *epg-5* mutant intestine. In the *epg-5* mutant, SMA-6::GFP and SNX-1::mCherry accumulate into larger punctate structures and partially colocalize. (**C-D**) Localization of MIG-14::GFP and SNX-3::mCherry in WT and *epg-5* mutant intestine. In the *epg-5* mutant, enlarged MIG-14::GFP and SNX-3::mCherry punctate structures partially colocalize. (**E-H**) Localization of H.s.TFR/hTfR::GFP, RFP::RAB-5, and mCherry::RAB-10 in WT and *epg-5* mutant intestine. WT intestine contains some small H.s.TFR/hTfR::GFP puncta that are distinct from RFP::RAB-5 (E) or mCherry::RAB-10 (G) puncta. In the *epg-5* mutant intestine, H.s.TFR/hTfR::GFP puncta accumulate and partially colocalize with enlarged RFP::RAB-5 (F) or mCherry::RAB-10 (H) punctate structures. (**I-K**) Quantification of colocalization of indicated markers in WT and *epg-5* mutant intestine (puncta ≥ 1μm in diameter were examined on five focal planes in at least three different animals). Scale bars: 10 μm. Inserts: 5 μm.


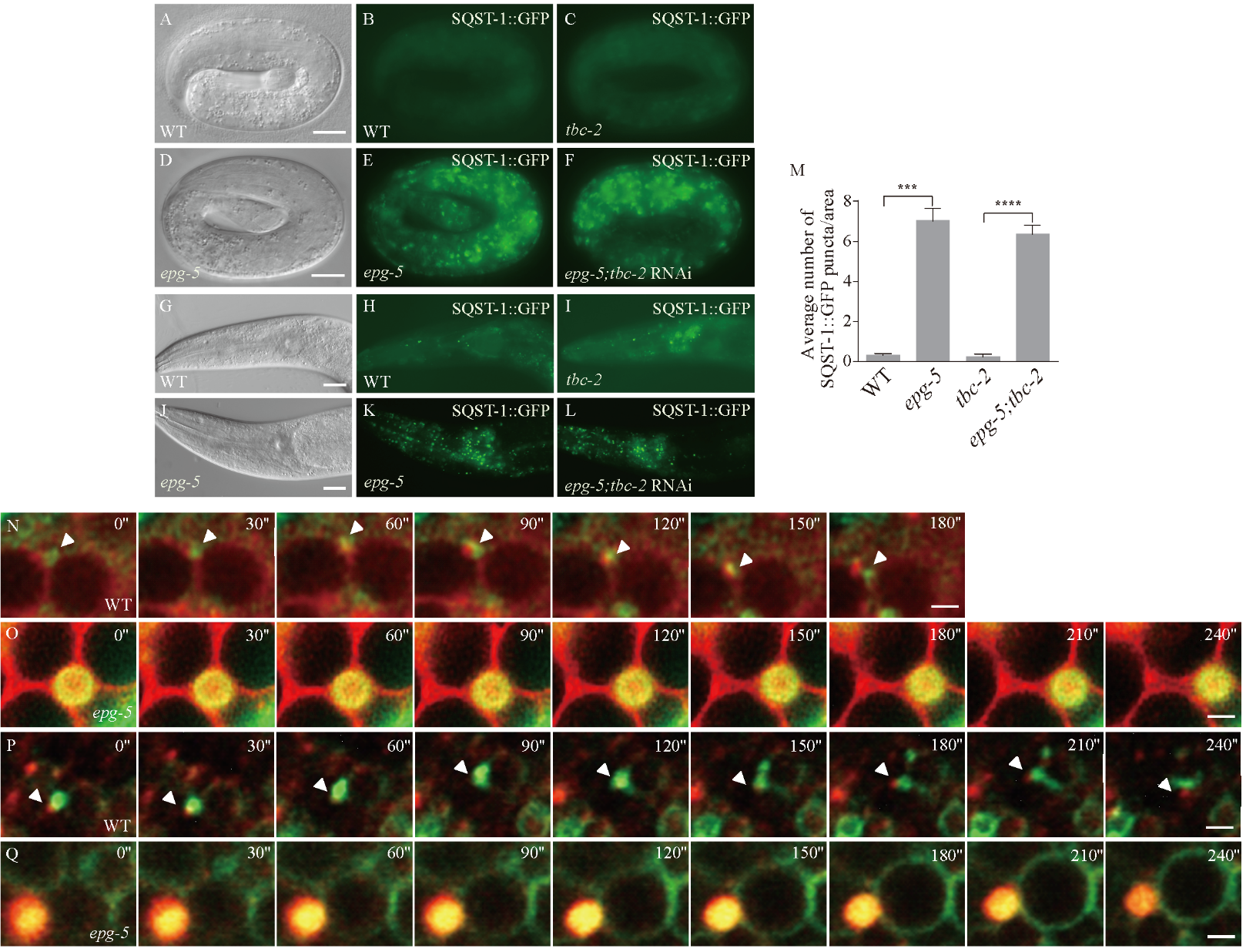


**Figure S4.** Knockdown of TBC-2 does not induce autophagic defects, related to Figure 5. (**A-F**) Localization of SQST-1::GFP in the embryonic stage of WT (A-B), *tbc-2* mutant (C), *epg-5* mutant (D-E), and *epg-5; tbc-2* RNAi worms (F). (**G-L**) Localization of SQST-1::GFP in the larval stage of WT (G-H), *tbc-2* mutant (I), *epg-5* mutant (J-K), and *epg-5; tbc-2* RNAi worms (L). DIC images of embryo and larvae (A,D,G,J). Scale bars: 10μm (A-F), 20μm (G-L). (**M**) Quantification of SQST-1::GFP puncta in pharynx of WT, *epg-5*, *tbc-2*, and *epg-5;tbc-2* mutants at larval stage. (**N-O**) Time-lapse of RFP::RAB-10 and GFP::RAB-5 in the intestine of wild type and *epg-5* mutants. White arrowheads indicate the change of GFP::RAB-5 location (N). (**P-Q**) Time-lapse of RFP::RAB-5 and GFP::RAB-7 in the intestine of WT and *epg-5* mutants. White arrowheads indicate the change of RFP::RAB-5 location (P). Scale bars: 1 μm.

**
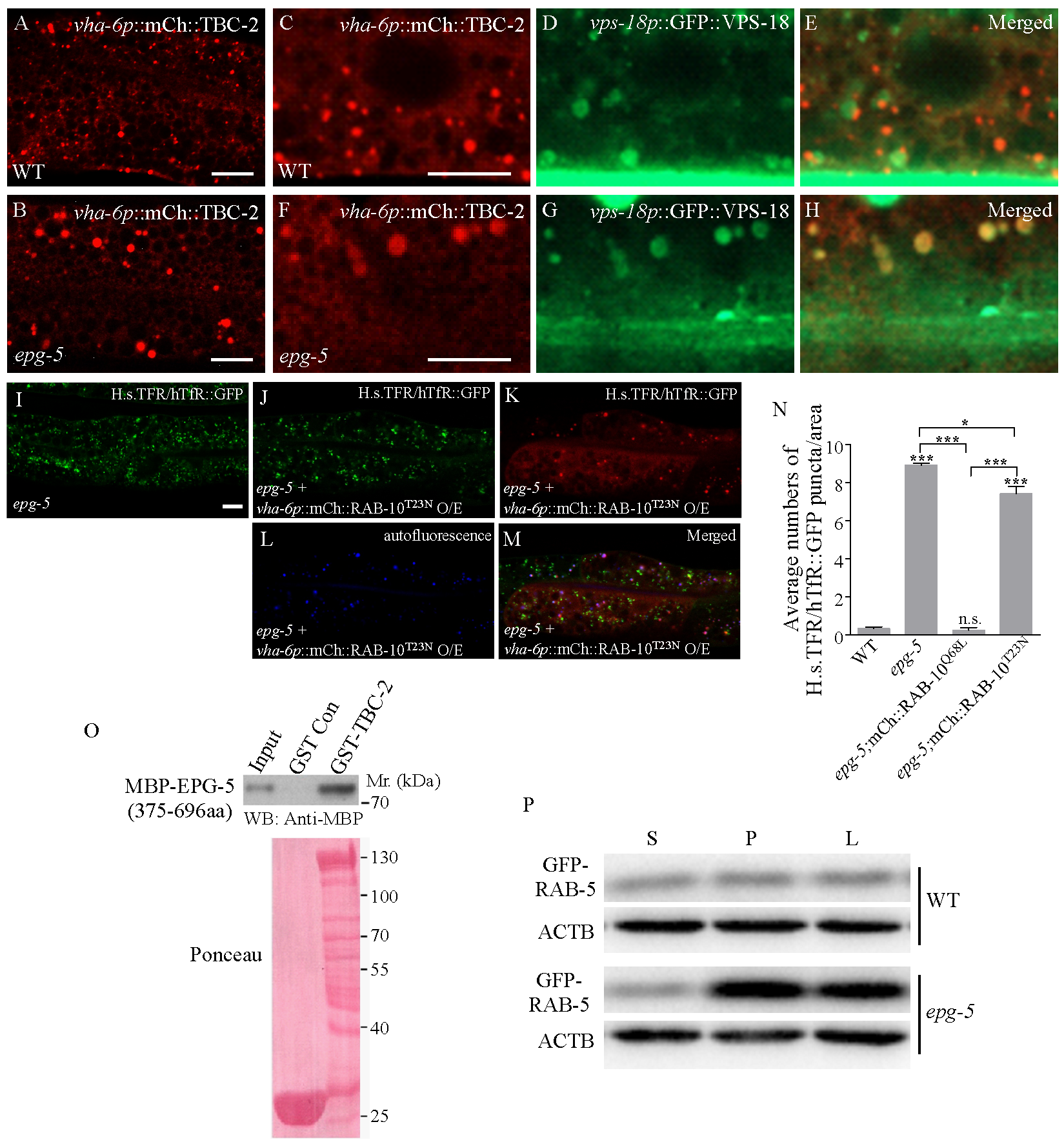
**

**Figure S5.** EPG-5 plays a role in the function of TBC-2. (**A-B**) Distribution of mCherry::TBC-2 in WT and *epg-5* mutant intestine. (**C-H**) Localization of mCherry::TBC-2 and GFP::VPS-18 in WT (C-E) and *epg-5* mutant (F-H) intestine. In the *epg-5* mutant, the enlarged mCherry::TBC-2 and GFP::VPS-18 structures colocalize. (**I-M**) Overexpression of the dominant-negative RAB-10^T23N^ showed much less extent rescue of the endocytic trafficking defect of H.s.TFR/hTfR in the *epg-5* mutant intestine. Scale bars: 10 μm. (**N**) Quantification of numbers of H.s.TFR/hTfR puncta ( ≥ 1 μm in diameter) in WT, *epg-5* mutants, *epg-5* mutants with overexpression of RAB-10^Q68L^, or RAB-10^T23N^. (**O**) EPG-5 (375-696aa) binds to full-length TBC-2 in *in vitro* affinity-isolation assay. (**P**) The membrane-to-cytosol ratio of RAB-5 is increased in *epg-5* mutants. S: supernatants after ultra-centrifugation of worm lysates; P: pellets after ultra-centrifugation of worm lysates; L: worm lysates.


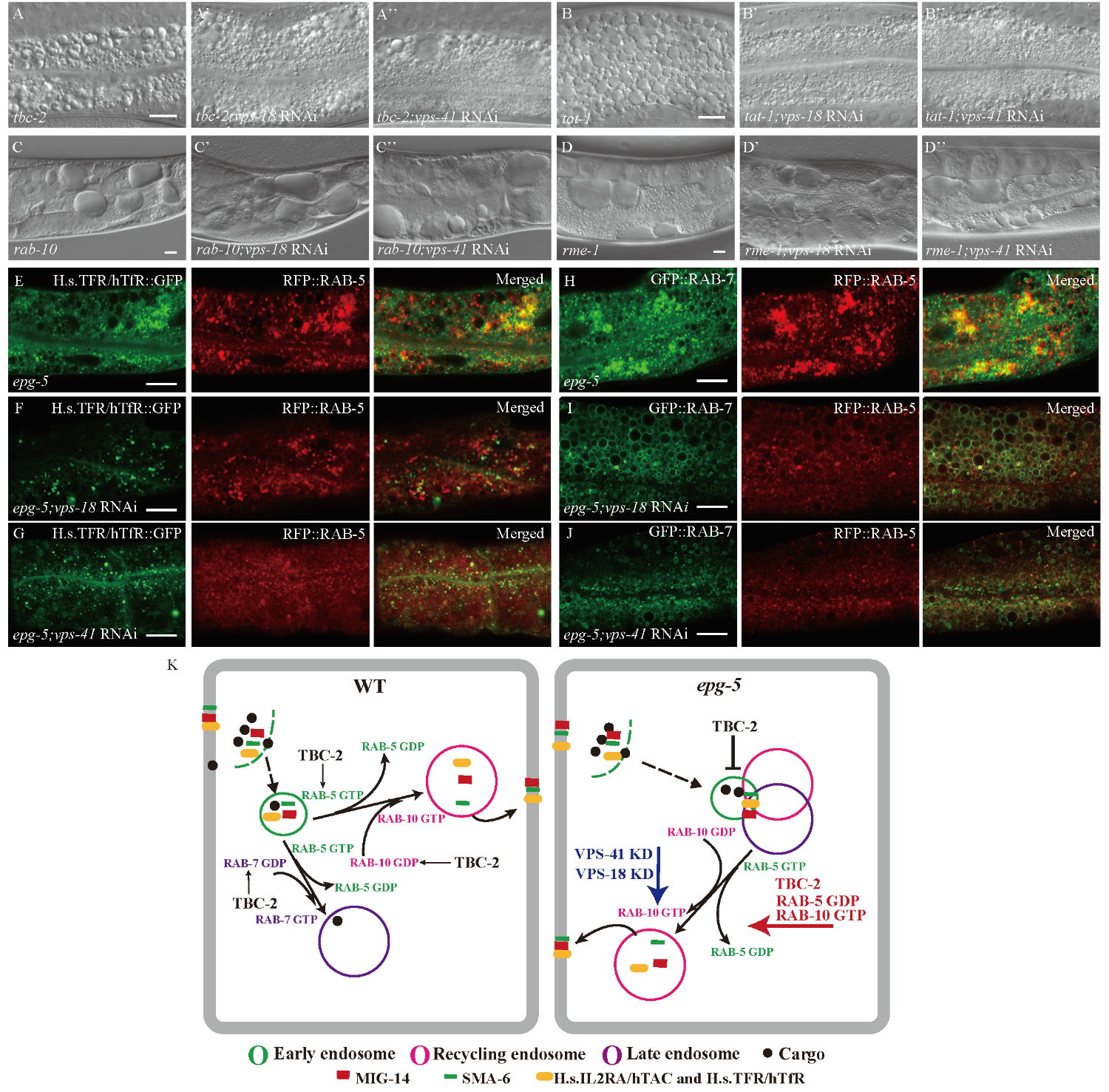


**Figure S6.** The HOPS complex is required for the intestinal vacuole formation in *tbc-2* or *tat-1* mutants, but not in *rab-10* or *rme-1* mutants, related to Figure 7. (**A-B’’**) DIC images show that *vps-18* RNAi or *vps-41* RNAi could suppress the formation of vacuoles in *tbc-2* mutant (A-A’’) and *tat-1* mutant (B-B’’) intestine. (**C-D’’**) DIC images show that *vps-18* RNAi or *vps-41* RNAi could not suppress the formation of vacuoles in *rab-10* mutant (C-C’’) or *rme-1* mutant (D-D’’) intestine. (**E-J**) *vps-18* RNAi or *vps-41* RNAi rescue the abnormal colocalization of hTfR::GFP with RFP::RAB-5 (E-G), and GFP::RAB-7 with RFP::RAB-5 (H-J) in *epg-5* mutants. Scale bars: 10 μm. (**K**) Proposed model showing the role of EPG-5 in membrane trafficking.
